# Sexual and reproductive health in Britain during the first year of the COVID-19 pandemic: cross-sectional population survey (Natsal-COVID-Wave 2) and national surveillance data

**DOI:** 10.1136/sextrans-2022-055680

**Published:** 2023-03-27

**Authors:** Kirstin R Mitchell, Malachi Willis, Emily Dema, Andrew J Baxter, Anne Connolly, Julie Riddell, Raquel Bosó Pérez, Soazig Clifton, Jo Gibbs, Clare Tanton, Rebecca Geary, Natasha Ratna, Hamish Mohammed, Magnus Unemo, Christopher Bonell, Andrew Copas, Pam Sonnenberg, Catherine H Mercer, Nigel Field

**Affiliations:** 1 MRC/CSO Social and Public Health Sciences Unit, University of Glasgow, Glasgow, UK; 2 Institute for Global Health, University College London, London, UK; 3 NatCen Social Research, London, UK; 4 Public Health and Policy, London School of Hygiene and Tropical Medicine, London, UK; 5 Institute of Population Health, University of Liverpool, Liverpool, Merseyside, UK; 6 Blood Safety, Hepatitis, Sexually Transmitted Infections (STI) and HIV Division, UK Health Security Agency, London, UK; 7 Department of Laboratory Medicine, Örebro University Hospital, Örebro, Sweden

**Keywords:** COVID-19, SEXUAL BEHAVIOUR, SEXUAL HEALTH, REPRODUCTIVE HEALTH, PREGNANCY, SEXUALLY TRANSMITTED INFECTIONS, CERVICAL SCREENING, SEXUAL DIFFICULTIES, SEXUAL SATISFACTION

## Abstract

**Objectives:**

To assess sexual behaviour, and sexual and reproductive health (SRH) outcomes, after 1 year of the COVID-19 pandemic in Britain.

**Methods:**

6658 participants aged 18–59 and resident in Britain completed a cross-sectional web-panel survey (Natsal-COVID-Wave 2, March-April 2021), 1 year after the first lockdown. Natsal-COVID-2 follows the Natsal-COVID-Wave 1 survey (July-August 2020) which captured impacts in the initial months. Quota-based sampling and weighting resulted in a quasi-representative population sample. Data were contextualised with reference to the most recent probability sample population data (Natsal-3; collected 2010–12; 15 162 participants aged 16–74) and national surveillance data on recorded sexually transmitted infection (STI) testing, conceptions, and abortions in England/Wales (2010–2020). The main outcomes were: sexual behaviour; SRH service use; pregnancy, abortion and fertility management; sexual dissatisfaction, distress and difficulties.

**Results:**

In the year from the first lockdown, over two-thirds of participants reported one or more sexual partners (women 71.8%; men 69.9%), while fewer than 20.0% reported a new partner (women 10.4%; men 16.8%). Median occasions of sex per month was two. Compared with 2010–12 (Natsal-3), we found less sexual risk behaviour (lower reporting of multiple partners, new partners, and new condomless partners), including among younger participants and those reporting same-sex behaviour. One in 10 women reported a pregnancy; pregnancies were fewer than in 2010–12 and less likely to be scored as unplanned. 19.3% of women and 22.8% of men were distressed or worried about their sex life, significantly more than in 2010–12. Compared with surveillance trends from 2010 to 2019, we found lower than expected use of STI-related services and HIV testing, lower levels of chlamydia testing, and fewer conceptions and abortions.

**Conclusions:**

Our findings are consistent with significant changes in sexual behaviour, SRH, and service uptake in the year following the first lockdown in Britain. These data are foundational to SRH recovery and policy planning.

**WHAT IS ALREADY KNOWN ON THIS TOPIC**

- Early in the pandemic, studies suggested a reduction in sexual risk behaviour, a decline in sexual frequency and desire, and an increase in virtual activities for some.
- The pandemic also significantly affected access to SRH services, as well as to preventive and reproductive technologies.

**WHAT THIS STUDY ADDS**

- This study shows that reductions in sexual risk behaviour and service uptake detected early in the pandemic were still evident 1 year following the first COVID-19 lockdown in Britain.
- This study also suggests that after 1 year there were fewer reported pregnancies, fewer reported abortions, and increased sexual dissatisfaction and distress, compared with what might be expected based on previous survey and surveillance data.

**HOW THIS STUDY MIGHT AFFECT RESEARCH, PRACTICE OR POLICY**

- These data suggest that recovery should focus on restoring STI prevention behaviours, provision of free or low-cost condoms, catching-up on service-provision backlogs, counselling for sexual difficulties, and sex education for young people who missed out during the pandemic.

## Introduction

Sexual and reproductive health (SRH) is vital for population well-being.^1^ Services like detection and management of sexually transmitted infections (STIs), fertility management and pregnancy-related care are continually required, even during lockdowns.^2 3^ In Britain, national COVID-19 lockdowns began on 23 March 2020 and on 5 January 2021, each lasting approximately 4 months, interspersed by fluctuating restrictions. SRH services in Britain were severely disrupted. Although positive innovations occurred—including telemedicine and self-administered at-home mifepristone/misoprostol for early abortion care—the overall picture suggested reduced or suspended services.^2 4 5^ Evidence indicated that people self-censured their sexual health needs and experienced barriers to service access^3^; in the 4 months after the first lockdown began, 9.7% of people in Britain reported at least one failed attempt to access an SRH service.^2^ Surveillance data showed a large reduction in sexual health clinic attendance and STI/HIV testing in April-May 2020, which was only partially offset by increased online testing. Clinic attendance and STI/HIV testing increased through the second half of 2020 but did not reach pre-pandemic levels.^6^ Prescribing of HIV pre-exposure prophylaxis (PrEP) and long-acting reversible contraception (LARC) also fell and only partially recovered.^7^

During the pandemic’s early months, changes in sexual behaviour were primarily due to reduced opportunities to have sex for people not cohabiting with a partner. While many people reported no changes, most studies found that frequency of partnered sex declined overall.^8^ Young people and those not in cohabiting relationships were more likely to report decreased sexual frequency and satisfaction and increased non-partnered activities like masturbation and viewing pornography.^9 10^ These groups and men who have sex with men (MSM) reported fewer sexual partners,^11^ and risk reductions in MSM were commonly found across studies.^8^ In steady relationships, people commonly reported improved relationship quality but diminished sex life quality.^12^ Studies variously reported no change, improvement, and declines in sexual function, with declines more pronounced for women than men.^8^ However, evidence on population sexual behaviour and SRH is weak; most initial studies used convenience sampling and lacked a baseline, preventing assessment of the pandemic’s impact. The timeframe for these studies was also too short to reliably detect changes in SRH outcomes, such as pregnancy and abortion. Thus, early reviews have called for longer-term evidence.^8^

The Natsal COVID-19 study was conducted in two waves. Natsal-COVID-1 (first wave) was conducted 4 months after the first UK lockdown (July-August 2020) and provided population-level evidence on behaviour and service use in the initial months.^2 3 9 12^ Here we present data from the second wave (Natsal-COVID-2; March-April 2021), which was designed to track behaviour over a longer period and provide 1 year estimates of SRH outcomes. We examined patterns of sexual behaviour, SRH service use, pregnancy, abortion and fertility management, and sexual function and sex life quality in the year following the first lockdown.

## Methods

### Study design

The second wave survey (Natsal-COVID-2) was undertaken between 27 March and 26 April 2021, approximately 1 year after the first UK lockdown, using similar methods to the first survey (undertaken between 29 July and 10 August, 4 months after first lockdown). Detailed methods are presented elsewhere.^13 14^ Data were collected using a 13 min online questionnaire (available at https://www.natsal.ac.uk/natsal-covid-study) through a web-panel survey administered by Ipsos MORI. Participants provided informed consent via an online form before starting the survey.

### Participants and procedures

The target core sample was 6000 people aged 18–59 years, with an additional boost of 500 people aged 18–29 years. To achieve a quasi-representative sample of the British population, we used quotas of age, gender, region, and social grade. Data were subsequently weighted to match the expected population distributions for the quota characteristics and sexual identity. The sample was drawn first from participants taking part in the first survey and agreeing to re-contact (n=5535), of whom 2098 completed the second survey (longitudinal analyses will be reported elsewhere). No quotas were set for this group. To reach the target sample, new participants were sampled until the quotas were reached. We obtained ethical approval from the University of Glasgow (20019174) and the London School of Hygiene and Tropical Medicine (LSHTM) (22565). Table 1 defines key terms describing participants.

**Table 1:** Definition of Key terms in Natsal-COVID-2

Term	Definition
Sexually experienced	Natsal-COVID-2: Reporting any lifetime sexual partner contact (vaginal, anal, oral sex or other genital contact) (Natsal-3: reporting sex with at least one person over the lifetime (vaginal, oral or anal sex))
Women/men/all	‘Women’ and ‘men’ each include trans women and trans men, respectively, but not those who identify in another way. The denominator for cervical cancer screening and reproductive health outcomes is participants described as female at birth, which includes cis women, trans men, and some non-binary participants. ‘All’ includes men, women and those who identify in another way
Women who have sex with women (WSW)/men who have sex with men (MSM)	Reporting sex with at least one person of the same sex in the past 5 years. We acknowledge that these groups are heterogeneous in behaviours and risks, and they include individuals reporting exclusively same-sex partners as well as those with same-sex and opposite-sex partners

In Natsal-COVID-2, 67 participants reported their sex at birth as different to their gender identity and were classified as ‘trans’, including 26 trans men, 19 trans women, and 22 who identified in another way (includes non-binary participants).

### Comparison with pre-COVID population data and surveillance data trends

**Figure 1:**
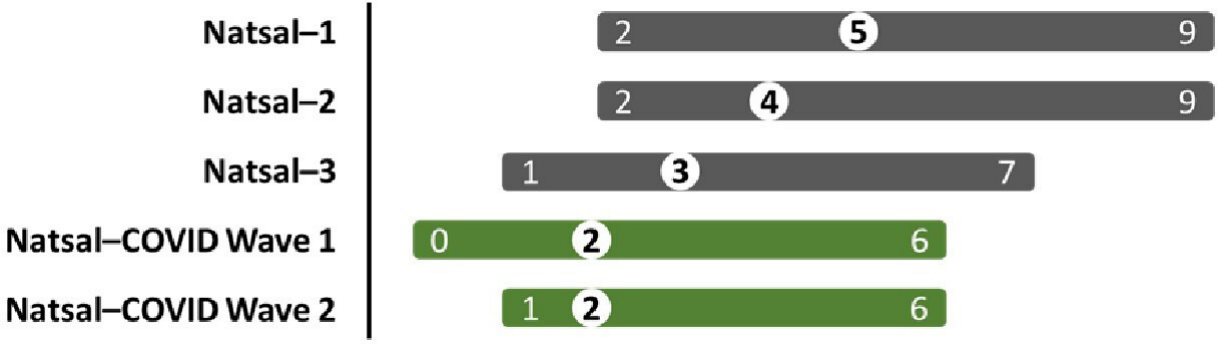
Median occasions of sex in the past 4 weeks across Natsal surveys. Median (central number in white circle) and interquartile range (numbers at ends of bars) for occasions of sex in the past 4 weeks are depicted for Natsal-1 (1990–1991), Natsal-2 (1999–2001), Natsal-3 (2010–2012), Natsal-COVID-1 (2020), and Natsal-COVID-2 (2021). To enable comparison across surveys, the age range was restricted to 18–44 years to match the ages of participants in Natsal-2. The denominator is people of any gender. Occasions of sex include oral, vaginal, anal sex or other genital contact.

There is no available pre-pandemic population baseline on SRH as such data are not routinely collected. To assess the extent to which observed outcomes might be due to the pandemic we compared our findings with data from Natsal-3, the most recent national survey (data collected 2010–12; 15 162 participants aged 16–74)).^15 16^ Natsal-3 used a multistage, clustered, and stratified probability sample design conducting face-to-face interviews in participants’ homes. Natsal-1 (1990) and Natsal-2 (2000) used broadly similar methods (figure 1). We also examined trends in national surveillance data during the decade between Natsal-3 (2010–12) and 2019 to assess how secular trends might have contributed to measured differences between the surveys, and we present data points for 2020 where available (see data sources in table 2). Surveillance data observations from 2010 to 2019 represent an ‘unexposed population’. Data points from 2020 include events during the first pandemic year and possible anticipatory effects in pre-lockdown months; thus 2020 data points cannot be used to estimate reliably covid-exposed or covid-unexposed rates and are not included in trend analysis. Surveillance data do not capture underlying sexual behaviours or unmet need in the general population^17^ and may themselves be subject to impacts of the pandemic on data collection, but they indicate year-on-year trends not captured in survey data, and are not subject to selection and reporting bias.

**Table 2:** Data sources for national surveillance data

Variables (as displayed in text/figures)	Source
Conceptions per 100 women in England and Wales	Office of National Statistics (ONS). Conceptions in England and Wales, 2019. Release date: 5 August 2021 (www.ons.gov.uk)
Abortions per 100 women in England and Wales	Department of Health and Social Care (DHSC). Abortion statistics, England and Wales: 2020. Updated 4 January (www.gov.uk/government/statistics/)
Chlamydia testing, HIV testing, and sexual health clinical STI-related attendance rates per 100 people in England	Data from UK Health Security Agency (UKHSA, formerly Public Health England (PHE)) Sources: https://www.eurosurveillance.org/content/10.2807/1560-7917.ES2014.19.48.20981 https://www.gov.uk/guidance/gumcad-sti-surveillance-system https://www.gov.uk/guidance/ctad-chlamydia-surveillance-system
Cervical cancer screening (estimated uptake among women in the same age groups in a comparable sample)	Cervical Screening Programme, England 2018-19 (National Statistics). NHS Digital. https://digital.nhs.uk/data-and-information/publications/statistical/cervical-screening-annual/england---2018-19 (accessed 17 March 2022). Calculation of expected number of women attending screening: % of all women **invited** in a single year (all women divided by intervals in between screening) Women aged 25–49, screened every 3.5 years: 28.6% Women aged 50–59, screened every 5.5 years: 18.2% Average **coverage** 70% **Weighted number** of women in each age group in analysis: Women aged 25–49: 1992 Women aged 50–59: 818 **Estimated number** of Natsal-COVID-2 women invited to screen in 1 year (# x % invited): Women aged 25–49 (1992×0.286): 569.712 Women aged 50–59 (818×0.182): 148.876 Total (569.712+148.876) = 718.588 **% in Natsal-COVID-2 sample expected to be invited** in 1 year (total invited/all aged 25–59): 718.588/2810 = 25.57% invited % **expected uptake** (% invited x 0.7) 25.57% x 0.7=**17.9%** expected uptake

Notes. 1. We used data sources from England or England and Wales as a reasonable proxy for Britain (86.7% of participants in Natsal-COVID-2 survey resided in England). 2. Pregnancy and abortion rates were recalculated from published rates to include only women aged 18 or over. Chlamydia testing, HIV testing and clinic attendance included women and men aged 18–44.

STI, sexually transmitted infection.

### Statistical analyses

**Figure 2:**
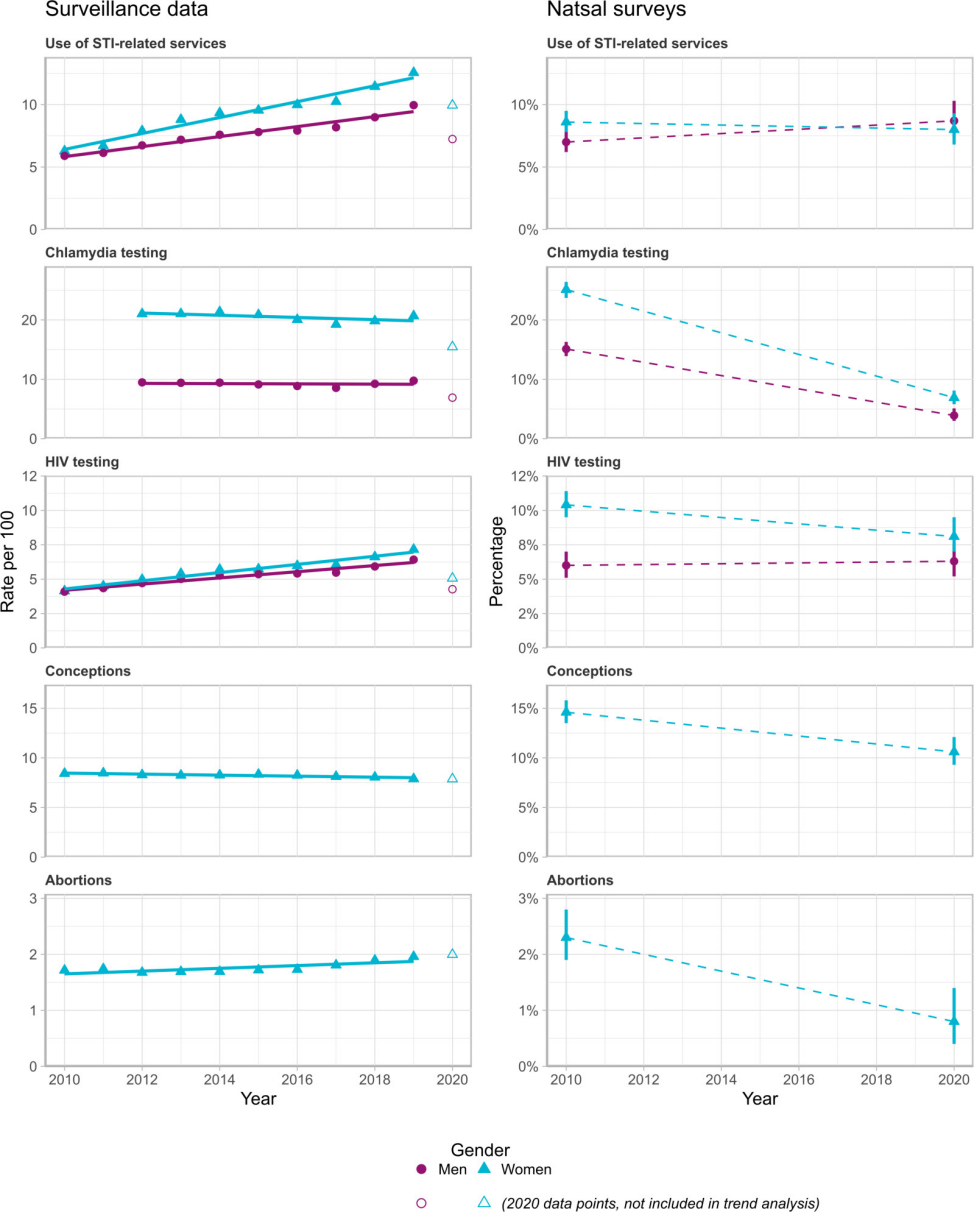
Comparison of annual surveillance data for sexual and reproductive health outcomes, 2010 to 2020, with equivalent outcomes in Natsal-3 (2010–2012) and Natsal-COVID-2 (2021). Chlamydia testing surveillance data are sourced from sexual health services’ and community-based settings’ routine returns to the GUMCAD STI Surveillance System and CTAD Chlamydia Surveillance System (UK Health Security Agency (UKHSA)). HIV testing and clinic attendance surveillance data are sourced from routine sexual health services’ returns to the GUMCAD STI Surveillance System. Clinic attendance surveillance data are restricted to sexually transmitted infection (STI)-related attendances only. Surveillance data are reported as counts of events per 100 persons. Natsal survey data are presented as percentages of participants who reported at least one event. We used dates up to 2019 to visualise baseline trends. Data points for 2020 were not used for trend analyses as they include pre- and post-pandemic events. Surveillance rates of conceptions and abortions include all women aged 18 and over. Chlamydia testing, HIV testing and clinic attendance rates include women and men aged 18–44.

Outcome variables are described in online supplemental box 3. Data are presented for participants aged 18–59 years, with exceptions stated. We used Stata’s (version 16.1) complex survey functions to incorporate weighting and stratification. Figure 2 was constructed in R (v4.2.1) using ‘ggplot2’.^18 19^ We present descriptive statistics for reported outcome variables by age group, gender, and same-sex behaviour. We tested differences in selected risk behaviours by gender and same sex behaviour and calculated age-adjusted odds ratios (aOR) using logistic regression. Where available for both surveys, we report aORs for outcome variables in Natsal-COVID-2 vs Natsal-3. Item non-response in Natsal-COVID-2 was typically 1–4% for the variables in this analysis. For surveillance data, we plotted annual rates, inspected for pre-covid linear trends (2010–19) and fitted linear models.

10.1136/sextrans-2022-055680.supp1 Supplementary data

## Results

A total of 6658 participants (women 49.9%, men 49.8%, identified in another way 0.4%) completed the Natsal-COVID-2 survey; 92.2% reported sexual experience ever.

### Patterns of sexual behaviour

Among sexually experienced participants aged 18–59 years, 71.8% of women and 69.9% of men reported at least one sexual partner in the year starting from the first lockdown; 35.5% of women and 37.0% of men reported having sex at least once per week in the preceding 4 weeks; this proportion declined with age among both women (from 41.3% (age 18–24) to 24.3% (age 45–59) and men (from 48.2% to 26.0%) (table 3). The median reported occasions of sex per month was two, compared with medians of three in 2010 (Natsal-3), four in 2000 (Natsal-2), and five in 1990 (Natsal-1) (figure 1).

**Table 3:** Patterns of sexual behaviour in Britain during the first year of the COVID-19 pandemic

Women	18–24 years	25–29 years	30–34 years	35–44 years	45–59 years	All*	All Natsal-3	aOR‡	All WSW†	All Natsal-3 WSW	aOR
Occasions of sex, past 4 weeks Median (IQR)	2 (0 to 6)	3 (1 to 6)	3 (1 to 6)	2 (0 to 5)	2 (0 to 4)	2 (0 to 5)	3 (1 to 6)	–	2 (0 to 6)	3 (1 to 6)	–
Frequency of vaginal, anal, or oral sex, past 4 months*											
Not in past 4 months	21.6 (17.5 to 26.4)	18.4 (15.1 to 22.3)	19.4 (15.8 to 23.7)	29.7 (26.3 to 33.4)	42.5 (39.4 to 45.6)	30.8 (29.1 to 32.6)	–	–	22.4 (15.7 to 30.9)	–	–
Less than once a week	37.1 (32.0 to 42.5)	32.0 (27.9 to 36.4)	35.0 (30.3 to 40.0)	33.3 (29.8 to 37.0)	33.2 (30.3 to 36.3)	33.7 (31.9 to 35.5)	–	–	38.4 (29.8 to 47.9)	–	–
At least once a week	41.3 (36.1 to 46.7)	49.6 (45.0 to 54.1)	45.0 (40.6 to 50.7)	37.0 (33.4 to 40.7)	24.3 (21.7 to 27.2)	35.5 (33.7 to 37.3)	–	–	39.2 (30.4 to 48.6)	–	–
Number of sexual partners, past year											
0 partners	28.5 (23.7 to 33.8)	20.6 (17.0 to 24.6)	19.3 (15.6 to 23.6)	27.0 (23.6 to 30.6)	34.4 (31.5 to 37.5)	28.2 (26.6 to 30.0)	15.9 (14.9 to 16.9)	–	18.9 (12.5 to 27.6)	4.4 (2.4 to 8.0)	–
1 partner	53.8 (48.2 to 59.2)	68.4 (63.8 to 72.6)	75.7 (71.1 to 79.8)	68.8 (65.1 to 72.3)	64.9 (61.9 to 67.9)	66.4 (64.6 to 68.2)	70.6 (69.4 to 71.9)	0.35 (0.31 to 0.40); p<0.0001	54.9 (45.4 to 64.0)	47.7 (41.3 to 54.0)	0.19 (0.09 to 0.44); p=0.0001
2+ partners	17.8 (13.9 to 22.5)	11.1 (8.3 to 14.5)	4.9 (3.1 to 7.8)	4.2 (2.9 to 6.1)	0.6 (0.3 to 1.5)	5.4 (4.6 to 6.3)	13.5 (12.6 to 14.4)	0.30 (0.25 to 0.36); p<0.0001	26.2 (18.6 to 35.6)	47.9 (41.6 to 54.3)	0.38 (0.23 to 0.64); p=0.0002
Number of new sexual partners, past year											
0 partners	67.6 (62.2 to 72.5)	83.1 (79.3 to 86.4)	89.0 (85.3 to 91.9)	91.0 (88.5 to 93.0)	97.5 (96.2 to 98.3)	89.6 (88.4 to 90.7)	81.9 (80.9 to 82.9)	–	62.8 (53.0 to 71.6)	56.4 (50.1 to 62.6)	–
1 partner	22.9 (18.6 to 27.9)	11.4 (8.7 to 14.7)	8.5 (6.1 to 11.9)	7.4 (5.6 to 9.7)	2.4 (1.6 to 3.7)	7.8 (6.9 to 8.9)	11.5 (10.7 to 12.3)	0.44 (0.38 to 0.51); p<0.0001	22.1 (14.7 to 31.8)	19.4 (15.1 to 24.5)	0.80 (0.50 to 1.29); p=0.36
2+ partners	9.5 (6.7 to 13.4)	5.5 (3.7 to 8.2)	2.4 (1.2 to 4.7)	1.6 (0.9 to 2.9)	0.1 (0.0 to 0.8)	2.5 (2.0 to 3.2)	6.6 (6.1 to 7.3)	0.31 (0.23 to 0.40); p<0.0001	15.2 (9.7 to 22.9)	24.2 (19.2 to 30.0)	0.59 (0.32 to 1.08); p=0.091
Number of new condomless sexual partners, past year§											
0 partners	80.6 (75.8 to 84.7)	88.3 (84.9 to 91.1)	93.3 (90.2 to 95.5)	94.8 (92.7 to 96.2)	98.5 (97.5 to 99.1)	93.6 (92.6 to 94.4)	–	–	90.9 (83.0 to 95.4)	–	–
1 partner	14.8 (11.2 to 19.3)	9.4 (7.0 to 12.6)	5.6 (3.6 to 8.6)	4.2 (2.9 to 6.1)	1.5 (0.9 to 2.5)	5.2 (4.4 to 6.1)	–	–	5.7 (2.2 to 13.8)	–	–
2+ partners	4.6 (2.8 to 7.6)	2.3 (1.2 to 4.2)	1.1 (0.5 to 2.9)	1.0 (0.5 to 2.1)	0.0	1.2 (0.9 to 1.7)	–	–	3.4 (1.4 to 8.1)	–	–
Denominator (unweighted, weighted)	390, 311	515, 414	415, 343	719, 659	1012, 1128	3051, 2856	6469, 5432	–	165.80	331 230	–

Men	18–24 years	25–29 years	30–34 years	35–44 years	45–59 years	All*	All Natsal-3	aOR‡	All MSM†	All Natsal-3 MSM	aOR
Occasions of sex, past 4 weeks Median (IQR)	2 (1 to 8)	3 (1 to 7)	3 (1 to 7)	2 (1 to 5)	2 (0 to 4)	2 (0 to 6)	3 (1 to 6)	–	2 (0 to 5)	3 (1 to 6)	–
Frequency of vaginal, anal, or oral sex, past 4 months*											
Not in past 4 months	20.0 (15.7 to 25.1)	19.5 (15.3 to 24.4)	21.1 (16.0 to 27.3)	25.2 (21.8 to 29.0)	43.4 (40.2 to 46.6)	30.2 (28.3 to 32.1)	–	–	16.2 (11.3 to 22.6)	–	–
Less than once a week	31.7 (26.5 to 37.5)	30.3 (25.1 to 35.9)	33.3 (27.0 to 40.3)	37.3 (33.4 to 41.4)	30.6 (27.7 to 33.7)	32.8 (30.9 to 34.8)	–	–	45.0 (37.0 to 53.3)	–	–
At least once a week	48.2 (42.4 to 54.1)	50.3 (44.5 to 56.0)	45.5 (38.6 to 52.6)	37.4 (33.6 to 41.4)	26.0 (23.3 to 29.0)	37.0 (35.0 to 39.0)	–	–	38.8 (31.0 to 47.3)	–	–
Number of sexual partners, past year											
0 partners	34.3 (28.8 to 40.3)	31.3 (26.0 to 37.2)	21.3 (16.1 to 27.7)	22.5 (19.2 to 26.1)	35.7 (32.6 to 38.8)	30.0 (28.2 to 32.0)	11.5 (10.5 to 12.5)	–	16.2 (11.3 to 22.6)	3.9 (1.8 to 8.2)	–
1 partner	41.2 (35.3 to 47.4)	50.0 (44.0 to 56.0)	68.9 (62.0 to 75.1)	69.8 (65.9 to 73.5)	61.1 (57.9 to 64.2)	60.3 (58.2 to 62.4)	69.1 (67.6 to 70.5)	0.23 (0.20 to 0.27); p<0.0001	45.0 (37.0 to 53.3)	35.2 (27.5 to 43.8)	0.20 (0.08 to 0.51); p=0.0009
2+ partners	24.5 (19.5 to 30.3)	18.7 (14.3 to 24.1)	9.7 (6.2 to 14.9)	7.6 (5.8 to 10.1)	3.2 (2.2 to 4.5)	9.6 (8.4 to 11.0)	19.4 (18.3 to 20.7)	0.37 (0.31 to 0.43); p<0.0001	38.8 (31.0 to 47.3)	60.8 (52.2 to 68.8)	0.38 (0.24 to 0.62); p=0.0001
Number of new sexual partners, past year											
0 partners	61.5 (55.2 to 67.3)	71.9 (66.2 to 77.1)	80.3 (73.9 to 85.3)	84.2 (80.7 to 87.1)	93.9 (92.0 to 95.3)	83.2 (81.5 to 84.8)	76.7 (75.4 to 77.9)	–	52.4 (44.0 to 60.6)	43.3 (34.9 to 52.1)	–
1 partner	20.8 (16.2 to 26.1)	14.7 (11.1 to 9.3)	13.2 (9.1 to 18.8)	12.1 (9.4 to 15.4)	4.7 (3.4 to 6.4)	10.8 (9.5 to 12.2)	12.5 (11.6 to 13.5)	0.55 (0.48 to 0.64); p<0.0001	19.6 (13.9 to 27.0)	14.8 (10.1 to 21.1)	0.57 (0.35 to 0.92); p=0.022
2+ partners	17.8 (13.4 to 23.3)	13.3 (9.5 to 18.3)	6.5 (3.8 to 11.1)	3.7 (2.5 to 5.5)	1.5 (0.9 to 2.6)	6.1 (5.1 to 7.2)	10.8 (9.9 to 11.7)	0.46 (0.37 to 0.56); p<0.0001	28.0 (20.8 to 36.5)	41.9 (33.6 to 50.7)	0.50 (0.30 to 0.83); p=0.0075
Number of new condomless sexual partners, past year§											
0 partners	75.2 (69.7 to 80.0)	78.8 (73.3 to 83.4)	87.5 (81.9 to 91.6)	88.5 (85.3 to 91.1)	96.2 (94.6 to 97.3)	88.5 (87.1 to 89.9)	–	–	61.4 (52.7 to 69.5)	–	–
1 partner	14.8 (11.0 to 19.5)	12.2 (8.9 to 16.5)	9.7 (6.2 to 15.1)	9.4 (7.0 to 12.5)	3.1 (2.1 to 4.6)	8.0 (6.9 to 9.3)	–	–	18.1 (12.2 to 25.9)	–	–
2+ partners	10.1 (7.1 to 14.2)	9.1 (5.9 to 13.7)	2.7 (1.2 to 6.1)	2.1 (1.2 to 3.6)	0.7 (0.4 to 1.5)	3.5 (2.8 to 4.3)	–	–	20.5 (14.1 to 28.9)	–	–
Denominator (unweighted, weighted)	334, 371	329, 343	228, 271	679, 781	1112, 1103	2682, 2871	4847, 5899	–	262, 138	176, 177	–

Weighted percentages reported with 95% CIs in parentheses.

*‘All’ comprises sexually experienced participants aged 18–59 years, including women who have sex with women (WSW) and men who have sex with men (MSM).

†WSW and MSM were defined as women and men, respectively, who reported a previous same-sex experience in the past 5 years.

‡Age-adjusted odds ratios (aOR) compare 1 year prevalence rates in Natsal-COVID-2 vs Natsal-3. For each aOR, ‘0 partners’ is the reference group for a dichotomous outcome variable. As such, the aOR for ‘one partner’ represents odds of having one or more partners in the past year in Natsal-COVID-2 vs Natsal-3. Denominators for number of partners are shown; other denominators were of similar magnitude.

§Natsal-3 comparison not available due to lack of variable in Natsal-3 dataset.

There were significant gender differences in reported sexual behaviours associated with elevated STI risk. Fewer women (5.4%) than men (9.6%) reported two or more partners in the past year (aOR 0.51, 95% CI 0.41 to 0.63). Women were also less likely to report one or more new sexual partners (10.4% vs 16.8%; aOR 0.51, 95% CI 0.43 to 0.61) and one or more new condomless partners (6.4% vs 11.5%; aOR 0.48, 95% CI 0.39 to 0.59) in the past year (table 3). Among women and men, reporting of risk behaviours declined with age. Women who reported sex with women (WSW) and men who reported sex with men (MSM) were more likely to report STI risk behaviours than all women and all men, respectively (excepting condomless sex for WSW). Reporting these risk behaviours decreased with age.

Compared with 10 years previously (Natsal-3), women and men in Natsal-COVID-2 were less likely to report two or more sexual partners in the past year (women: 5.4% vs 13.5%, aOR 0.30, 95% CI 0.25 to 0.36; men: 9.6% vs 19.4%, aOR 0.37, 95% CI 0.31 to 0.43). Similar differences between surveys were observed for numbers of reported new sexual partners for women and men, and for WSW and MSM.

### SRH service use

To understand uptake of sexual health services during the pandemic, sexually experienced participants aged 18–44 years reported their use of STI-related services, chlamydia testing, HIV testing, and cervical cancer screening (table 4).

**Table 4:** Use of sexual health services, HIV and chlamydia testing, and cervical cancer screening in Britain during the first year of the COVID-19 pandemic

Women	18–24 years	25–29 years	30–34 years	35–44 years	45–59 years	All*	All Natsal-3‡	aOR†	All WSW	All Natsal-3 WSW	aOR
STI-related service use, past year	19.1 (15.1 to 24.0)	11.0 (8.3 to 14.3)	7.2 (4.9 to 10.5)	2.3 (1.5 to 3.7)	–	8.4 (7.1 to 9.8)	8.6 (7.8 to 9.5)	0.96 (0.77 to 1.18); p=0.67	–	–	–
Chlamydia test, past year	15.6 (12.1 to 20.0)	10.7 (8.1 to 13.8)	4.9 (3.2 to 7.5)	2.4 (1.5 to 3.9)	–	7.3 (6.2 to 8.5)	25.1 (23.7 to 26.4)	0.20 (0.16 to 0.24); p<0.0001	–	–	–
HIV test, past year	12.2 (9.0 to 16.4)	11.8 (9.0 to 15.3)	9.1 (6.6 to 12.4)	4.6 (3.2 to 6.6)	–	8.6 (7.4 to 10.0)	10.4 (9.5 to 11.4)	0.80 (0.66 to 0.99); p=0.029	–	–	–
Cervical cancer screening, past year§	–	16.3 (13.4 to 19.8)	12.4 (9.7 to 15.9)	11.2 (9.1 to 13.8)	6.7 (5.3 to 8.4)	10.3 (9.2 to 11.5)	–	–	–	–	–
Denominator (unweighted, weighted)	371, 290	518, 416	402, 330	706, 647	–	1997, 1683	4701, 3331	–	–	–	–

Men	18–24 years	25–29 years	30–34 years	35–44 years	45–59 years	All*	All Natsal-3‡	aOR†	All MSM	All Natsal-3 MSM	aOR
STI-related service use, past year	16.0 (12.2 to 20.8)	13.0 (9.6 to 17.5)	8.0 (4.9 to 12.8)	4.0 (2.8 to 5.7)	–	9.0 (7.6 to 10.6)	7.0 (6.2 to 7.9)	1.32 (1.04 to 1.67); p=0.023	36.2 (27.2 to 46.3)	27.0 (19.1 to 36.5)	1.55 (0.85 to 2.84); p=0.15
Chlamydia test, past year	7.0 (4.4 to 10.9)	5.0 (3.0 to 8.3)	5.0 (2.7 to 9.1)	2.0 (1.2 to 3.1)	–	4.1 (3.2 to 5.3)	15.1 (13.9 to 16.3)	0.21 (0.15 to 0.29); p<0.0001	17.3 (11.5 to 25.3)	35.1 (26.2 to 45.1)	0.39 (0.21 to 0.73); p=0.0034
HIV test, past year	8.6 (5.9 to 12.4)	7.8 (5.3 to 11.4)	9.4 (6.0 to 14.3)	4.0 (2.8 to 5.6)	–	6.5 (5.4 to 7.9)	6.0 (5.1 to 7.0)	1.10 (0.85 to 1.44); p=0.46	29.1 (21.1 to 38.7)	27.9 (19.7 to 38.0)	1.06 (0.56 to 1.98); p=0.86
Denominator (unweighted, weighted)	307, 342	326, 336	226, 264	652, 744	–	1511, 1686	3198, 3366	–	183, 114	126, 109	–

Weighted percentages reported with 95% CIs in parentheses.

*‘All’ comprises sexually experienced participants aged 18–44 years (25–59 for cervical cancer screening only), including WSW and MSM.

†Age-adjusted odds ratios (aOR) compare 1 year prevalence rates in Natsal-COVID-2 with Natsal-3. Data are presented as STI-related service use for Natsal-COVID-2, including STI testing, STI follow-up care, and HIV testing;.

‡Natsal-3 comparisons present sexual health clinic attendance and should therefore be interpreted with caution.

§Due to differences in ages represented, cervical cancer screening denominators are: 25–59 (582, 473); 30–34 (463, 385); 35–44 (816, 762); 45–59 (1088, 1217); All (2949, 2837); Natsal-3 comparison not available due to lack of variable in Natsal-3 dataset or differences between surveys regarding reporting timeframes.

MSM, men who reported sex with men; STI, sexually transmitted infection; WSW, women who reported sex with women.

In Natsal-COVID-2, reported use of STI-related services in the past year was highest among participants aged 18–24 years (women 19.1%; men 16.0%) and MSM of any age (36.2%). Men in Natsal-COVID-2 were more likely to report using STI-related services than men in Natsal-3 (aOR 1.32, 95% CI 1.04 to 1.67), but there were no clear differences for women (aOR 0.96, 95% CI 0.77 to 1.18), and the higher odds for MSM were not significant (aOR 1.55, 95% CI 0.85 to 2.84) (table 4).

Participants aged 18–44 years in Natsal-COVID-2 (women 7.3%; men 4.1%; MSM 17.3%) were less likely than those in Natsal-3 (women 25.1%; men 15.1%; MSM 35.1%) to report a chlamydia test in the past year (women: aOR 0.20, 95% CI 0.16 to 0.24; men: aOR 0.21, 95% CI 0.15 to 0.29; MSM: aOR 0.39, 95% CI 0.21 to 0.73). Differences between Natsal-COVID-2 and Natsal-3 were also present in young people and those reporting at least one new partner in the past year. For example, only 16.0% of participants reporting at least one new partner in Natsal-COVID-2 reported a chlamydia test in the past year, whereas this was 38.7% in Natsal-3 (data not shown).

HIV testing in the past year was reported by 8.6% of women, 6.5% of men, and 29.1% of MSM in Natsal-COVID-2 (sexually experienced participants). These proportions paralleled Natsal-3 for men and MSM, but women in Natsal-COVID-2 were less likely than women in Natsal-3 to report an HIV test in the past year (aOR 0.80, 95% CI 0.66 to 0.99) (table 4).

Among eligible participants aged 25–59 years, 10.3% reported cervical cancer screening in the past year (approximately 18% would be expected to report this based on uptake in a comparable 1 year period before the pandemic (see calculation, table 2).^20^ There was a strong association with age; those aged 45–59 years were less likely to report cervical cancer screening than those aged 25–29 years.

Surveillance data (figure 2) for use of STI services and HIV testing indicate linear increasing trends between 2010 and 2019, suggesting that 2020 rates would have been higher than 2010 rates if there had been no pandemic. Chlamydia testing rates appear stable in surveillance data, suggesting that 2020 rates might also have been stable without the pandemic. Against this backdrop, the comparison of survey data between Natsal-3 and Natsal-COVID-2 surveys and the 2020 surveillance data points are consistent in suggesting that the pandemic contributed to lower-than-expected rates for these outcomes

### Pregnancy, abortion and fertility management

Among sexually experienced participants described as female at birth and aged 18–44 years, 10.6% reported that they stopped using or switched their contraceptive method in the past year due to the pandemic; this was highest in those aged 18–24 years (16.4%) (table 5). Compared with Natsal-3, fewer participants reported a pregnancy in the past year (10.6% vs 14.6%; aOR 0.56, 95% CI 0.47 to 0.67). Pregnancies in Natsal-COVID-2 were less likely to be scored as unplanned (6.2% vs 18.3%; aOR 0.33, 95% CI 0.18 to 0.59). The proportion of participants who reported abortion in the past year was lower in Natsal-COVID-2 than in Natsal-3 (0.8% vs 2.3%; aOR 0.34, 95% CI 0.18 to 0.63).

**Table 5:** Pregnancy, abortion and fertility management in Britain during the first year of the COVID-19 pandemic

Described female at birth	18–24 years	25–29 years	30–34 years	35–44 years	All^*^	All Natsal-3	aOR
Pregnancy, past year	9.5 (6.8 to 13.3)	13.4 (10.6 to 16.8)	13.2 (10.2 to 17.1)	8.1 (6.3 to 10.3)	10.6 (9.3 to 12.1)	14.6 (13.5 to 15.8)	0.56 (0.47 to 0.67); p<0.0001
Unplanned	18.5 (8.4 to 35.9)	5.5 (2.0 to 13.9)	–	5.2 (1.8 to 13.9)	6.2 (3.6 to 10.3)	18.3 (15.3 to 21.7)	0.33 (0.18 to 0.59); p=0.0002
Ambivalent	57.3 (39.6 to 73.3)	30.5 (19.9 to 43.8)	24.8 (15.2 to 37.7)	35.6 (24.4 to 48.5)	34.9 (28.6 to 41.8)	26.9 (23.5 to 30.7)	–
Planned	24.2 (12.2 to 42.3)	64.0 (51.0 to 75.3)	75.2 (62.3 to 84.8)	59.2 (46.3 to 71.0)	58.9 (52.0 to 65.5)	54.8 (50.6 to 58.9)	–
Terminated pregnancy, past year	1.8 (0.7 to 4.5)	0.5 (0.2 to 1.7)	0.8 (0.3 to 2.6)	0.5 (0.1 to 1.9)	0.8 (0.4 to 1.4)	2.3 (1.9 to 2.8)	0.34 (0.18 to 0.63); p=0.0005
Stopped/switched contraceptive method, past year*	16.4 (12.7 to 20.9)	12.6 (9.9 to 15.9)	11.7 (8.8 to 15.4)	6.0 (4.4 to 8.1)	10.6 (9.3 to 12.1)	–	–
Denominator (unweighted, weighted)	408, 327	538, 437	429, 354	754, 694	2129, 1812	4252, 2959	–

Weighted percentages reported with 95% CIs in parentheses.

*‘All’ comprises sexually experienced women aged 18–44 years, including WSW.

†Age-adjusted odds ratios (aOR) compare 1 year prevalence rates in Natsal-COVID-2 with Natsal-3. The aOR for six-item London Measure of Unplanned Pregnancy (LMUP) scores in Natsal-Covid-2 and Natsal-3 represents odds of scoring 0–3 (unplanned) with the reference group being scores from 4 to 12 (ambivalent or planned).^28^ Denominators for pregnancy are shown; other denominators were of similar magnitude.

‡Natsal-3 comparison not available due to lack of variable in Natsal-3 dataset.

WSW, women who reported sex with women.

Surveillance data (figure 2) show a shallow decreasing trend in conceptions between 2010 to 2019 and an increasing trend in abortions, which compare with substantial falls in the estimates for both outcomes between Natsal-3 and Natsal-COVID-2. The surveillance data point for abortions in 2020—which includes pre-lockdown abortions—is in line with baseline trends.

### Sexual dissatisfaction, distress and difficulties

Sexual dissatisfaction and distress about sex were common experiences in the first year of the pandemic. Dissatisfaction increased with age (from 19.7% (age 18–24) to 28.6% (age 45–59) among women, and from 17.0% to 41.5% among men) (table 6). In contrast, levels of distress were similar across age groups among women, and decreased among men (from 26.5% to 18.5%). Women were less likely than men to report dissatisfaction and distress (26.8% vs 32.2%; aOR 0.77, 95% CI 0.68 to 0.87 for dissatisfaction, and 19.3% vs 22.8%; aOR 0.81, 95% CI 0.71 to 0.93 for distress). There was no significant variation in these outcomes by reported same-sex behaviour, except that MSM were more likely to report distress than all men (37.7% vs 22.8%; aOR 1.95, 95% 1.35 to 2.81).

**Table 6:** Sexual function and sex life quality in Britain during the first year of the COVID-19 pandemic

Women	18–24 years	25–29 years	30–34 years	35–44 years	45–59 years	All*	All Natsal-3	aOR†	All WSW	All Natsal-3 WSW	aOR
Dissatisfied with sex life, past year	19.7 (15.9 to 24.2)	22.3 (18.7 to 26.4)	27.5 (23.3 to 32.2)	29.8 (26.4 to 33.3)	28.6 (25.9 to 31.5)	26.8 (25.2 to 28.5)	15.2 (14.2 to 16.2)	2.10 (1.88 to 2.36); p<0.0001	24.0 (17.4 to 32.3)	16.3 (78.3 to 88.0)	1.66 (0.97 to 2.85); p=0.066
Distressed with sex life, past year	21.5 (17.5 to 26.2)	27.4 (23.5 to 31.7)	21.6 (17.8 to 26.0)	20.5 (17.6 to 23.6)	14.2 (12.2 to 16.6)	19.3 (17.9 to 20.8)	11.4 (10.5 to 12.3)	1.82 (1.60 to 2.07); p<0.0001	28.5 (20.7 to 37.7)	12.9 (9.0 to 18.1)	2.71 (1.52 to 4.82); p=0.0007
Sexual difficulties, past year§	12.4 (9.4 to 16.3)	13.4 (10.6 to 16.8)	9.8 (7.2 to 13.2)	9.1 (7.1 to 11.5)	12.1 (10.1 to 14.3)	11.3 (10.2 to 12.6)	–	–	12.7 (7.7 to 20.2)	–	–
Avoided sex due to difficulties, past year§	11.9 (8.8 to 15.9)	11.8 (9.1 to 15.2)	9.3 (6.8 to 12.6)	8.9 (7.0 to 11.2)	11.8 (9.9 to 14.0)	10.8 (9.7 to 12.0)	–	–	13.5 (8.1 to 21.5)	–	–
Overall sex life in past year worse than previous year§	26.6 (22.2 to 31.6)	26.3 (22.5 to 30.4)	26.8 (22.6 to 31.5)	26.0 (22.8 to 29.4)	24.2 (21.6 to 27.0)	25.5 (23.9 to 27.2)	–	–	29.3 (21.8 to 38.0)	–	–
Denominator (unweighted, weighted)‡	411, 327	541, 429	429, 356	751, 693	1031, 1149	3163, 2960	6527, 5493	–	167, 82	341, 237	–

Men	18–24 years	25–29 years	30–34 years	35–44 years	45–59 years	All*	All Natsal-3	aOR†	All MSM	All Natsal-3 MSM	aOR
Dissatisfied with sex life, past year	17.0 (13.2 to 21.5)	21.0 (16.8 to 25.9)	22.8 (17.6 to 29.0)	35.5 (31.7 to 39.6)	41.5 (38.4 to 44.7)	32.2 (30.4 to 34.2)	18.1 (16.9 to 19.4)	2.34 (2.07 to 2.65); p<0.0001	27.1 (21.0 to 34.2)	25.8 (18.6 to 34.5)	1.43 (0.82 to 2.48); p=0.21
Distressed with sex life, past year	26.5 (21.7 to 31.9)	28.8 (23.9 to 34.3)	21.8 (16.5 to 28.1)	24.7 (21.2 to 28.5)	18.5 (16.1 to 21.1)	22.8 (21.1 to 24.6)	10.5 (9.5 to 11.5)	2.52 (2.19 to 2.89); p<0.0001	37.7 (30.0 to 46.0)	12.5 (7.9 to 19.1)	4.54 (2.55 to 8.09); p<0.0001
Sexual difficulties, past year§	20.0 (15.8 to 24.9)	17.7 (13.6 to 22.7)	8.7 (5.5 to 13.6)	9.7 (7.6 to 12.3)	6.1 (4.7 to 7.8)	10.6 (9.4 to 12.0)	–	–	29.5 (22.2 to 37.9)	–	–
Avoided sex due to difficulties, past year§	22.0 (17.6 to 27.3)	14.7 (11.0 to 19.3)	7.6 (4.7 to 12.1)	8.1 (6.1 to 10.6)	5.2 (3.9 to 6.9)	9.6 (8.5 to 10.9)	–	–	26.3 (19.2 to 34.9)	–	–
Overall sex life in past year worse than previous year§	18.7 (14.6 to 23.6)	22.6 (18.2 to 27.7)	22.5 (17.3 to 28.7)	31.1 (27.3 to 35.2)	27.9 (25.1 to 30.8)	26.3 (24.6 to 28.2)	–	–	29.4 (22.4 to 37.6)	–	–
Denominator (unweighted, weighted)‡	364, 407	361, 375	243, 287	709, 816	1143, 1132	2820, 3017	4891, 5953	–	263, 139	185, 187	–

Weighted percentages reported with 95% CIs in parentheses.

*‘All’ comprises sexually experienced participants aged 18–59 years, including WSW and MSM.

†Age-adjusted odds ratios (aOR) compare 1 year prevalence rates in Natsal-COVID-2 with Natsal-3.

‡Denominators for dissatisfied with sex life are shown; other denominators were of similar magnitude.

§Natsal-3 comparison not available due to lack of variable in Natsal-3 dataset or differences between surveys regarding question wording and response options.

MSM, men who reported sex with men; WSW, women who reported sex with women.

Overall, participants in Natsal-COVID-2 were more likely than those in Natsal-3 to report dissatisfaction (women: aOR 2.10, 95% CI 1.88 to 2.36; men: aOR 2.34, 95% CI 2.07 to 2.65) and distress (women: aOR 1.82, 95% CI 1.60 to 2.07; men: aOR 2.52, 95% CI 2.19 to 2.89). MSM in Natsal-COVID-2 were no more likely to report dissatisfaction than MSM in Natsal-3 (aOR 1.43, 95% CI 0.82 to 2.48), but were much more likely to report distress (aOR 4.54, 95% CI 2.55 to 8.09). Trends for WSW were similar to all women.

Around one in 10 sexually experienced participants reported sexual difficulties, and a similar proportion reported avoiding sex because of difficulties in the first year of the pandemic (table 6). For both outcomes, we observed decreases with age among men (from 20.0% (age 18–24) to 6.1% (age 45–59) for sexual difficulties; from 22.0% to 5.2% for avoiding sex), but not women. Both were more commonly experienced by MSM than all men (sexual difficulties: 29.5%, aOR 3.42, 95% CI 2.26 to 5.20; avoiding sex: 26.3%, aOR 3.15, 95% CI 2.01 to 4.93). There were no significant differences between WSW and all women (data not shown).

Participants compared their sex life during the first year of the COVID-19 pandemic with the previous year (table 6). Approximately one quarter (women: 25.5%, 95% CI 23.9% to 27.2%; men: 26.3%, 95% CI 24.6% to 28.2%) perceived their sex life during COVID-19 pandemic to be worse than the previous year; this increased with age for men (from 18.7% (age 18–24) to 27.9% (age 45–59)) but not women. There were no significant differences by gender or reported same-sex experience.

## Discussion

Fewer than 20% of participants reported a new partner during the first year of the pandemic, compared with more than 25% in Natsal-3 (2010–12). We observed much lower reported chlamydia testing in Natsal-COVID-2 than Natsal-3 (2010–2012). Sexual health service access and HIV testing were similar between the surveys, contrasting with rising trends suggested by surveillance data. Just under 10% of Natsal-COVID-2 participants described as female at birth reported that they switched or stopped using their contraception methods in the past year due to the pandemic. Pregnancies and abortions in the first year of the pandemic were lower than estimates from a decade earlier (Natsal-3). Surveillance data showing stable conception and rising abortion trends in the prior decade suggest that the pandemic contributed to reductions observed between the surveys. Distress and dissatisfaction with one’s sex life were common during the pandemic and at a significantly higher level than 10 years previously.

To our knowledge, Natsal-COVID-2 is the most comprehensive general population study of SRH during the COVID-19 pandemic.^8^ The study was not a probability sample but used quota-based sampling and weighting to improve generalisability. However, specific estimates should be interpreted with caution given likely selection and response biases. In particular, web-based surveys exclude people without digital access, who are more likely to be in lower social grades.^21^

Without a baseline, the best available comparison data source was the Natsal-3 survey, with two important caveats. First, the methodology was different. Natsal-3 was a household-based interviewer-led CAPI/CASI (computer-assisted personal interviewing/computer-assisted self-interviewing) probability sample, whereas Natsal-COVID-2 was a web-panel survey. Differential selection probabilities between surveys and other biases are only partially corrected by weighting.^22 23^ Second, Natsal-3 data were collected 10 years ago (2010–2012) and sexual behaviour, sexual mores and service provision have all undergone period changes, which affect comparisons. Methodological differences and secular change may partially account for the observed differences.

Frequency of sex has been steadily declining since Natsal-1 in 1990/91, and the decline observed in the present study may reflect a secular trend unrelated to the pandemic. Although we lack interim data for sexual behaviours, the rate of diagnosis for most STIs provides a proxy and has increased year-on-year in Britain during this period.^24^ Thus, but for the pandemic, we might have reasonably expected similar or increased levels of sexual risk behaviour compared with Natsal-3. The lower levels observed in our study were most pronounced in younger participants and men reporting same-sex behaviour—key populations with high rates of STIs. The data suggest a fall in the number of sexual partners, number of new partners and condomless sex with a new partner. This is consistent with what we know from studies earlier in the pandemic, including Natsal-COVID-1. Most of these earlier studies focused on MSM, finding a reduction in the number of casual partners and unprotected anal intercourse.^8^ These findings are intuitively plausible because lockdowns restricted physical and social interactions.^9 11^

Overall differences observed between Natsal-COVID-2 and Natsal-3 in chlamydia testing were also found in younger participants, who are targeted by the National Chlamydia Screening Programme (which, until June 2021, targeted opportunistic chlamydia testing to sexually active people under 25 years), and in those reporting at least one new partner in the past year, who are recommended to test by the British Association for Sexual Health and HIV (BASHH) guidelines. By contrast to the survey comparisons, surveillance data showed stable chlamydia testing and rising sexual health service attendance and HIV testing in the years preceding the pandemic. Taken together, we infer that the pandemic’s effect has been a significant drop in uptake of these services—a finding consistent with a large international study (I-SHARE).^25^ This occurred despite rapid responses by sexual health services (eg, online and telephone alternatives to face-to-face consultations).

Stopping or switching contraceptive methods is associated with a higher risk of unplanned pregnancy.^26^ Just under 10% of participants described as female at birth reported that they switched or stopped using their contraception methods in the past year specifically because of the pandemic. Evidence from other surveillance sources suggests periods of reduced capacity to provide LARC during the pandemic,^5 27^ and the pandemic itself might have exacerbated switching or stopping contraception. Pregnancies and abortions in the first year of the pandemic were lower than estimates from a decade earlier. Furthermore, the apparent fall in abortions may be greater since Natsal-COVID-2 employed a more direct question (‘have you ever had an abortion?’ prompting ‘Year of most recent abortion’), less prone to underreporting than the indirect measure used in Natsal-3 (‘Have you ever been pregnant?’, prompting ‘What was the outcome of that pregnancy?’ with ‘termination or abortion’ as possible response).^28^ Surveillance data showing stable conception and rising abortion trends in the prior decade suggest that the pandemic contributed to reductions observed between the surveys. The findings are consistent with a global review suggesting decreased access to abortion and reduced intention to get pregnant, especially for individuals facing housing and food insecurity.^29 30^ The greater reduction in unplanned compared with ambivalent or planned pregnancies might be due to fewer new partners and less casual sex.

Distress and dissatisfaction with one’s sex life were common during the pandemic, more so than 10 years previously in Natsal-3. The increased level of sexual distress and avoidance of sex among MSM in our study is striking. The finding that a quarter of Natsal-COVID-2 participants perceived their sex life during the pandemic to be worse than the previous year, adds to a suggested pandemic influence, and is supported by systematic review evidence suggesting an increase in sexual difficulties.^31^ Longitudinal tracking over the course of the pandemic is rare, though a study in Canada found no change in solitary desire and a decrease in dyadic desire between April and August 2020.^32^ Studies have linked declines in sexual satisfaction with being young, single or in a poor-quality relationship, and mental distress.^8^

While all the data sources we report here have limitations, they provide largely consistent evidence about the effects of the pandemic on SRH, which is supported by an emerging literature.^8^ Taken together, these data suggest COVID-19 had a significant influence on sexual and reproductive health, probably through a combination of restrictions on social mixing, disruption to SRH services, and pandemic-related uncertainty and stress. However, the longer-term implications are difficult to predict. It is unclear what might happen to STI incidence, not least because of uncertainties about sexual behaviours in the future, which might return to pre-pandemic levels or higher. Careful monitoring and research will be required. Similarly, disruptions to contraceptive uptake and pregnancy planning will be important to track, not only to safeguard individual control of fertility but to assess the sociodemographic impact of shifts in trend.

In recovery, it will be critical for public health messaging to emphasise safer sexual behaviour and STI testing. People may need reminding about the availability of free and confidential services, which in the UK include asymptomatic testing and PrEP. This is particularly important because some young people may have missed out on sex education due to school closure and disruption.^33^ It will be crucial to address intensified disparities in sexual and reproductive health arising from marginalised sexual and gender identities, especially where they intersect with ethnicity and poverty.^34^ The evidence suggests that detrimental impacts of the pandemic on sexual behaviour, function and mental health are closely intertwined,^8^ suggesting that an integrated approach to recovery is required.

## Data Availability

Data are available in a public, open access repository. Natsal-COVID-1 and Natsal-COVID-2 datasets are available at UK data archive (study #8865; 10.5255/UKDA-SN-8865-1 and DOI: 10.5255/UKDA-SN-8865-2 respectively)^35^
