# Supplementary material for: Sexual and reproductive health in Britain during the first year of the COVID-19 pandemic: cross-sectional population survey (Natsal-COVID-Wave 2) and national surveillance data
Source: Sex Transm Infect. 2023 Mar 27;99(6):386–97. doi: 10.1136/sextrans-2022-055680 (PMC10447381; doi:10.1136/sextrans-2022-055680)
Supplement: Supplementary data [file sextrans-2022-055680supp001.pdf]

BMJ Publishing Group Limited (BMJ) disclaims all liability and responsibility arising from any reliance placed on this supplemental material which has been supplied by the author(s)

SUPPLEMENTARY FILE

Box 3: Definitions of outcome variables

Outcome/variable	Definition
Occasions of sex, past four weeks	Sum of responses to 'On how many occasions in the last 4 weeks have you had sex with a woman,' '...with a man,' and 'with someone who was transgender or non-binary' (including oral, vaginal, anal sex or other genital contact)
Frequency of vaginal, anal, or oral sex, past four months	Reported engaging in vaginal, anal, or oral sex at least once a week in the past 4 months, less than once a week in the past 4 months, or not in the past 4 months
Number of sexual partners, past year	Number of people with whom participants reported sex (including oral, vaginal, or anal sex or any other contact involving the genital area), in the past year
Number of new sexual partners, past year	Number of new people with whom participants reported sex for the first time (including oral, vaginal, or anal sex with or any other contact involving the genital area), in the past year
Number of new condomless sexual partners, past year	Number of new people with whom participants reported vaginal or anal sex without using a condom on their first sexual encounter, in the past year
STI-related service use, past year	Natsal-COVID: Reported use of STI testing, STI follow-up care, or HIV testing services, in the past year.<br>Natsal-3: Reported sexual health clinic attendance, in the past year.
Chlamydia test, past year	Reported most recent chlamydia test in past year, derived from the following two questions: Have you ever been tested for chlamydia? If yes: When were you last tested for chlamydia?
HIV test, past year	Reported most recent HIV test in past year, derived from the following two questions: Have you ever had a test for HIV? If yes: When was your most recent HIV test?
Cervical Screening, past year	Reported use of cervical screening (smear test/pap test) health service, in the past year
Pregnancy, past year	Reported currently pregnant or pregnancy in the past year (even if the baby was not carried to term)
Pregnancy planning, past year	Six-item London Measure of Unplanned Pregnancy (LMUP)[26] to estimate the degree of planning among participants reporting a pregnancy in the past year; scores from 0–3 are categorised as unplanned, 4–9 as ambivalent, and 10–12 as planned
	Reported having an abortion (abortion medication or surgery), in

	the past year
Terminated pregnancy, past year	
Stopped/switched contraceptive method, past year	Reported at least one of the following occurring in the past year due to the pandemic: temporarily/permanently stopped using any method to prevent pregnancy, temporarily/permanently changed to a different method to prevent pregnancy
Dissatisfied with sex life, past year	‘Strongly disagree’ or ‘Disagree’ in response to ‘I feel satisfied with my sex life’ in the past year
Distress with sex life, past year	‘Strongly agree’ or ‘Agree’ in response to ‘I feel distressed or worried about my sex life’ in the past year
Sexual difficulties, past year	‘Very often’ or ‘Always’ in response to ‘Experience any sexual difficulty or difficulties’ (e.g., ‘anxiety, pain, vaginal dryness, difficulty getting an erection/aroused, difficulty reaching climax (orgasm) or reaching climax too soon’) in the past year
Avoiding sex, past year	‘Very often’ or ‘Always’ in response to ‘Avoid sex due to sexual difficulties’ in the past year
Deterioration in sex life, past year	‘Worse’ in response to ‘Compared to the year before the first lockdown, on the whole would you say that your sex life is better, about the same, or worse these days?”
Note. Data are presented for participants aged 18–59 years, except for reported use of STI-related services and reproductive health outcomes which are limited to those aged 18–44 years, due to small numbers in those older than 44 years or for cervical screening which was limited to those 25–59 years, reflecting age eligibility criteria for this intervention.	
